# OralExplorer: a web server for exploring the mechanisms of oral inflammatory diseases

**DOI:** 10.1186/s12967-024-05019-8

**Published:** 2024-03-15

**Authors:** Weiyin Lin, Hong Yang, Jiayu Lin, Xia Yang, Zhihao Liao, Yifan Zheng, Peng Luo, Chufeng Liu

**Affiliations:** 1 Stomatological Hospital, School of Stomatology, Southern Medical University, Guangzhou, China; 2 The Department of Oncology, Zhujiang Hospital, Southern Medical University, Guangzhou, China

**Keywords:** Oral disease, Shiny, Web tool, Bioinformatic analyses, Mechanism

## Abstract

**Background:**

Oral inflammatory diseases are localized infectious diseases primarily caused by oral pathogens with the potential for serious systemic complications. However, publicly available datasets for these diseases are underutilized. To address this issue, a web tool called OralExplorer was developed. This tool integrates the available data and provides comprehensive online bioinformatic analysis.

**Methods:**

Human oral inflammatory disease-related datasets were obtained from the GEO database and normalized using a standardized process. Transcriptome data were then subjected to differential gene expression analysis, immune infiltration analysis, correlation analysis, pathway enrichment analysis, and visualization. The single-cell sequencing data was visualized as cluster plot, feature plot, and heatmaps. The web platform was primarily built using Shiny. The biomarkers identified in OralExplorer were validated using local clinical samples through qPCR and IHC.

**Results:**

A total of 35 human oral inflammatory disease-related datasets, covering 6 main disease types and 901 samples, were included in the study to identify potential molecular signatures of the mechanisms of oral diseases. OralExplorer consists of 5 main analysis modules (differential gene expression analysis, immune infiltration analysis, correlation analysis, pathway enrichment analysis and single-cell analysis), with multiple visualization options. The platform offers a simple and intuitive interface, high-quality images for visualization, and detailed analysis results tables for easy access by users. Six markers (IL1β, SRGN, CXCR1, FGR, ARHGEF2, and PTAFR) were identified by OralExplorer. qPCR- and IHC-based experimental validation showed significantly higher levels of these genes in the periodontitis group.

**Conclusions:**

OralExplorer is a comprehensive analytical platform for oral inflammatory diseases. It allows users to interactively explore the molecular mechanisms underlying the action and regression of these diseases. It also aids dental researchers in unlocking the potential value of transcriptomics data related to oral diseases. OralExplorer can be accessed at https://smuonco.shinyapps.io/OralExplorer/ (Alternate URL: http://robinl-lab.com/OralExplorer).

**Supplementary Information:**

The online version contains supplementary material available at 10.1186/s12967-024-05019-8.

## Background

Oral inflammatory diseases comprise infectious diseases affecting both the soft and hard tissues of the oral cavity, including periodontitis, peri-implantitis, and caries. If left untreated, these diseases can lead to complications both in the maxillofacial and systematic area, such as cardiovascular diseases [1, 2], digestive diseases [3–6], diabetes [7], pulmonary diseases [8, 9], and neurological diseases [10–13]. Hence, early diagnosis and treatment of oral inflammatory diseases are vital for maintaining both oral and general health. However, the precise biological mechanisms underlying these diseases remain incompletely understood. To address this gap, researchers have turned to transcriptomics data, which allows a deeper exploration of the molecular-level biological mechanisms involved in oral inflammatory diseases. For instance, Song et al. [14] successfully identified genes (CTSS, PLEK, IRF-8, PTGS2, and FOSB) that may contribute to the development and progression of periodontitis through the analysis of transcriptome data from periodontitis samples as well as healthy samples. These findings offer new theoretical support for the diagnosis and prediction of periodontitis.

Although there are a wealth of oral disease-related datasets stored in publicly available databases, they are not sufficient to support the effective mining and utilization of these resources. For instance, the Gene Expression Omnibus (GEO) database contains a great number of user-uploaded datasets, including oral disease samples and normal oral samples, that can be freely downloaded. However, the extraction and analysis of the data heavily depend on programming or software processing, which the GEO platform itself does not provide. As a result, dental researchers without a programming background often struggle with fully utilizing the available data due to the lack of suitable data analysis tools. To address this issue, zero-code web tools that support online data analysis have emerged as a potential solution. In the oncology field, the TIMER web platform [15] is a typical example, offering a wide range of built-in datasets and the ability for users to upload their own data for comprehensive analyses of different cancer types and immune infiltration landscapes. Extensive research and analysis of publicly available datasets in the field of oral and maxillofacial diseases reveal a similar underutilization of data. Therefore, the development of an integrated online analysis platform with a wide range of built-in oral inflammatory disease datasets would greatly benefit dental researchers, enabling them to fully explore existing data, identify molecular characteristics of diseases, and further investigate disease-related mechanisms. This platform would provide new insights for clinicians in diagnosing and treating oral diseases.

Based on this context, we developed OralExplorer, a web-based tool that specifically targets the exploration of inflammatory diseases in the oral cavity. The primary objective of OralExplorer is to retrieve comprehensive datasets pertaining to oral inflammatory diseases from large public databases and to subsequently preprocess the data. This web tool will also enable users to conduct a wide array of bioinformatics analyses through our dedicated server. The primary purpose of OralExplorer is to facilitate the convenient mining of oral data resources, easy exploration of disease-related biomarkers, and provision of bioinformatics support for theoretical hypotheses about disease mechanisms.

## Methods

### Data collection

We conducted a search in the public GEO database for datasets pertaining to oral inflammatory diseases. As part of our screening process, we focused exclusively on datasets involving human subjects. We excluded any datasets that did not include a control group or lacked sufficient clinical information. Ultimately, we identified and included 35 datasets that were relevant to our study on oral inflammatory diseases (specifically periodontitis and peri-implantitis) (Additional file 1: Table S1). Additionally, we took the initiative to clean and integrate data from certain datasets that encompassed different stages of treatment for the same disease. These consolidated datasets were also incorporated into our research.

### Data preprocessing and analysis

Bulk RNA-seq data for all 27 datasets and their corresponding clinical information were obtained using the GEOquery package [16]. The transcriptomic data included high-throughput sequencing and microarray data. We applied Fragments Per Kilobase of transcript per Million mapped reads (FPKM) conversion to datasets that only had raw sequencing count reads to obtain uniform processing. All expression profiling data were manually verified for precompleted normalization. For those datasets without normalization, we used the limma package [17]. Gene symbol conversion was performed using the AnnoProbe package [18] and manually cross-checked. The eight single cell RNA-seq datasets were obtained from the GEO dataset website (https://www.ncbi.nlm.nih.gov/geo/). Seurat objects were then generated utilizing the Seurat package (version 4.4.0) [19]. Low-quality cells were eliminated by filtering out those with fewer than 200 or more than 2500 UMI counts and > 5% of mitochondrial genes. The resulting filtered data was used for subsequent analyses.

For the analysis of differential gene expression, we utilized the limma package to identify genes that were differentially expressed between the disease and normal groups. These differentially expressed genes were then ranked based on the magnitude of their log2FoldChange values. To visually represent the results of the gene differential expression analysis, we employed the EnhancedVolcano package [20] to create a volcano plot and the ComplexHeatmap package [21] to generate a heatmap.

For the immune infiltration analysis, we focused on the analysis results obtained from various immune infiltration algorithms. To achieve this, we utilized the IOBR package [22], which integrates multiple immune infiltration algorithms. The immune infiltration algorithms supported by IOBR include TIMER [15], xCell [23], CIBERSORT [24], EPIC [25], quanTIseq [26], and MCP-counter [27]. Nonimmunologically relevant cells identified by these algorithms were excluded, leaving us with a total of 42 immune cell types for further analysis (Additional file 2: Table S2). To compare the difference in immune infiltration between the disease and normal groups, we employed the Wilcoxon rank sum test. The visualization of the immune infiltration analysis involved the use of heatmaps, boxplots, and bubble plots. The implementation of these visualizations was performed with the ComplexHeatmap package, the ggpubr package [28], and the corrplot package [29].

For pathway enrichment analysis, Gene Set Enrichment Analysis (GSEA) and Single-sample Gene Set Enrichment Analysis (ssGSEA), were employed. Initially, a collection of 13,661 commonly used pathways from the Molecular Signatures Database (MSigDB) was obtained. This collection includes 50 hallmark pathways, 3050 C2 canonical pathways, and 10,561 C5 gene ontology pathways (Additional file 3: Table S3). The differential expression analysis results were subjected to GSEA using the clusterProfiler package [30]. The visualization of the results utilized the enrichplot package [31] and the GseaVis package [32]. Three types of visualization methods were used: dotplot, enrichmap, and enrichplot. ssGSEA was performed using the GSVA package [33]. The limma package was used to analyse the difference between the ssGSEA pathway enrichment scores of the disease groups and the control groups. Boxplots were generated using the ggpubr package, and heatmaps were plotted using the ComplexHeatmap package.

In the correlation analysis, our focus was on the correlations between different genes, between genes and immune infiltration, and between genes and pathways. We conducted a pairwise correlation study between different genes using two analysis algorithms: Spearman and Pearson. We utilized the ggplot2 package [34] to generate correlation scatterplots and the circlize package [35] to plot correlation scatter plots and multigene correlation chordal plots. Additionally, we calculated the correlation between gene expression levels and immune infiltration scores. We then visualized the results of their correlation analyses using the ggplot2 package as a heatmap. To examine the correlation between genes and pathways, we conducted a correlation analysis between ssgsea scores and gene expression values calculated using the GSVA package. The results were then visualized as correlation heatmaps using the ggplot2 package.

In the single-cell analysis, each dataset underwent normalization using the "NormalizeData" function, and the "vst" method was employed to identify the 2000 most variable features of each dataset through the "FindVariableFeatures" function. Subsequently, the data was scaled to adjust for sequencing depth using "ScaleData," and Principal component analysis (PCA) was conducted to cluster the cells initially via the "RunPCA" method. The determination of the number of principal components was customized for different datasets using an ElbowPlot. Following this, a K-nearest-neighbors plot, based on Euclidean distances in PCA space and utilizing the aforementioned principal component parameters, was constructed using "FindNeighbors." Clustering was further optimized by implementing the Louvain algorithm through the "FindClusters" function, which enhances the modularity of the dataset and combines cells based on global and local features. Subsequently, non-linear dimensionality reduction was performed using T-distributed Stochastic Neighbor Embedding (t-SNE) to facilitate dataset visualization and exploration. The "FindAllMarkers" function was then utilized to identify cell identity markers, with genes having a log-fold change threshold > 0.25 considered significant as differentially expressed genes (DEGs). Cell types were further annotated manually based on DEGs within different cells. The functions mentioned above are derived from the Seurat package. Additionally, ssGSEA scores were calculated for each cell and cluster using the GSVA package. The ssGSEA scores encompass 186 KEGG pathways, 1615 Reactome pathways, and 50 Hallmark pathways. The features of genes and ssGSEA scores were visualized using the "FeaturePlot" function of the Seurat package. Furthermore, the heatmap of genes and ssGSEA scores within each cluster was generated using the ComplexHeatmap package.

### Website development

The OralExplorer website was developed using R. The primary tool used for constructing the web interface and implementing interactive functionality was the Shiny package. OralExplorer aims to provide users with a user-friendly web interface that facilitates exploration of transcriptome biomarker analysis of oral disease datasets. The website comprises eight modules: Home, Input Data, Differential Gene, Immunoinfiltration, Correlation Analysis, Enrichment Analysis, Single-cell Analysis and Docs. We independently deployed OralExplorer on both the shinyapps.io platform (https://smuonco.shinyapps.io/OralExplorer/) and our local server (http://robinl-lab.com/OralExplorer) for free access.

### Gingival tissue sample collection

From September 2022 to October 2022, we collected samples from six subjects with moderate to severe periodontitis as well as six subjects with good periodontal health among patients treated at the Stomatological Hospital, School of Stomatology, Southern Medical University. The study protocols were approved by the Ethics Committee of Stomatological Hospital of Southern Medical University, and informed consent was obtained from all volunteers. Criteria for subject recruitment included the following: (i) voluntary completion of an informed consent form; (ii) diagnosis of vertical impaction by oral and maxillofacial surgery and recommendation for vertical impaction aiding eruption; (iii) patients with gingival hyperplastic lesions, moderate/severe chronic periodontitis diagnosed by periodontology, and gingival hypertrophy, hyperplasia, or the presence of pseudo periodontal pockets that are not conducive to plaque control after basic periodontal treatment requiring periodontal surgery; (iv) no sex restrictions; and (v) 18–50 years of age. The exclusion criteria were as follows: (i) the presence of underlying systemic diseases (e.g., heart disease, diabetes, hypertension, blood diseases) in patients who were unable to tolerate the corresponding surgery; (ii) pregnancy in women; and (iii) taking antibacterial or anti-inflammatory drugs in the past 3 months.

### Quantitative real-time PCR (qPCR)

After sampling, gingival tissue samples were stored in ep tubes without RNase, placed in liquid nitrogen and processed within 2 h. We extracted total RNA from gingival tissues using the TRIzol method (AG RNAex Pro RNA) according to the corresponding instructions, measured the concentration of the RNA precipitate after dissolving it in diethylcarbamoyl pyrocarbonate (DEPC) water, adjusted the mass of RNA for all samples to 1000 ng and reverse-transcribed the total RNA to cDNA in a quantitative polymerase chain reaction (qPCR) reaction system (Roche LoghtCycler 96, Roche, China) to set up the qPCR reaction programme. The gene expression of IL1β, SRGN, CXCR1, FGR, ARHGEF2 and PTAFR was analysed by the 2 - ΔΔCt method using β-actin as a control. Details of the primers used are shown in Table 1.

**Table 1:** Primers of target genes

Gene	Forward (5′-3′)	Reverse (5′-3′)
IL-1β	TTCGAGGCACAAGGCACAA	TGGCTGCTTCAGACACTTGAG
CXCR1	CTGACCCAGAAGCGTCACTTG	CCAGGACCTCATAGCAAACTG
PTAFR	ATGGAGCCACATGACTCCTC	AATGACCCCGAGCACAAAGAT
SRGN	ACTGACCTTTTTCCAAAGACGAG	CTGATCCAGAGTAGTCCTCAGAA
FGR	ACTATGAGGCTCGAACTGAGG	TCAGCTTGGATTGAGTCAACAG
ARHGEF2	CAGGCATGACCATGTGCTATG	TTTACAGCGGTTGTGGATAGTC

Details of the primers of target genes

### Immunohistochemistry (IHC)

After the extraction of gingival tissues, samples were fixed using 4% paraformaldehyde (ES-8100, ECOTOP, Guangzhou, China) for 24 h. The fixed samples were then dehydrated, embedded in paraffin, and sectioned into 4-μm thick slices. Immunohistochemical staining was employed to determine the localization of IL-1beta, SRGN, CXCR1, and PTAFR expression. First, the tissue sections were subjected to heat-induced epitope antigen retrieval in sodium citrate buffer (pH 6.0). Next, endogenous peroxidase activity was inactivated with 3% H2O2, and the sections were treated with 5% goat serum at room temperature to prevent nonspecific protein binding. The sections were then incubated with primary antibodies, including anti-IL-1beta antibody (1:800, GB11113-100, Servicebio, Wuhan, China), anti-SRGN antibody (1:100, A6951, ABclonal, Wuhan, China), anti-CXCR1 antibody (1:500, GB11625-100, Servicebio, Wuhan, China), and anti-PTAFR antibody (1:100, bs-1478R, Bioss Antibodies, Beijing, China) for 12 h at 4 °C. Following incubation with primary antibodies, the sections were washed with PBS. Subsequently, the sections were incubated with a secondary antibody for 50 min at room temperature. Colour development was achieved by adding 3,3′-diaminobenzidine (DAB) substrate, and the sections were counterstained with haematoxylin for better visualization.

All images of immunohistochemical results were obtained using a digital pathology scanner (Aperio VERSA) and analysed using ImageJ software. In brief, three immunohistochemical images of gingival tissues were randomly selected from both the healthy and inflammatory groups. These images were used to identify the specific area where the epithelial layer intersects with the lamina propria. Additionally, three fields of view were randomly chosen to calculate the area. ImageJ software was employed to process the images, specifically for colour deconvolution of DAB and haematoxylin staining. The resulting brown channel images captured DAB staining, which was suitable for further analysis. To ensure consistency, a uniform measurement threshold was applied to all images. The area fraction was then calculated by determining the ratio of the positively stained area to the area of the fixed rectangular box. Statistical analysis was carried out to assess the differences between the healthy and inflammatory groups.

### Statistical analysis

Statistical analysis was performed in the R software environment. Boxplots were used to display the data, with the median indicated by the centerline and the interquartile spacing represented by the boxes on either side. Bivariate differences were assessed for statistical significance using the Wilcoxon rank sum test. Two-sided tests were conducted to calculate p values, and values less than 0.05 were considered statistically significant. Asterisks were used to indicate the level of significance based on the following p values: *: p < 0.05, **: p < 0.01, ***: p < 0.001, ****: p < 0.0001.

## Results

**Fig. 1:**
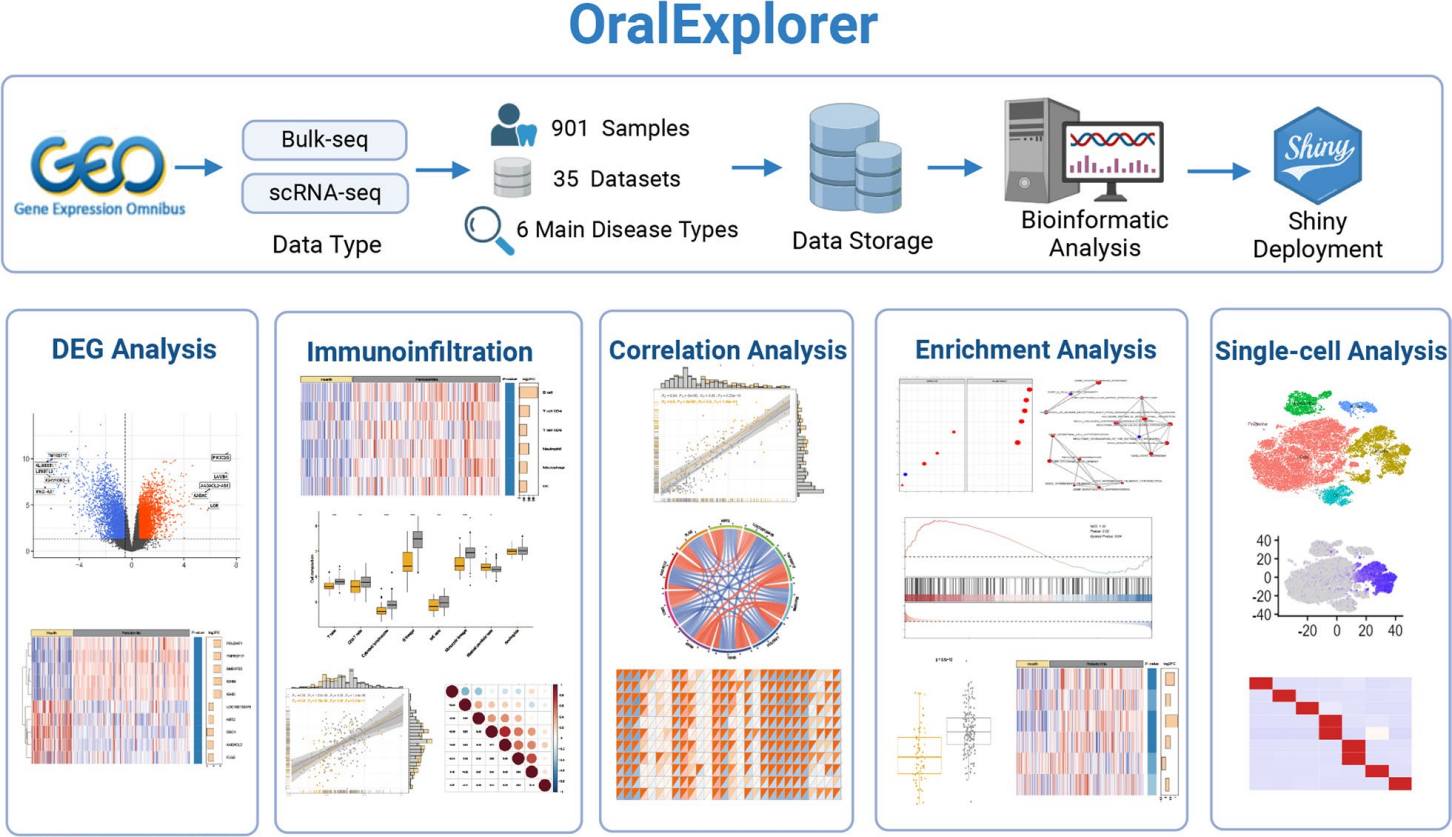
Overview of the data processing flow and analysis modules of OralExplorer. OralExplorer collects oral inflammatory disease data from the GEO database and performs subsequent analysis and web tool construction. OralExplorer consists of five major modules: differential gene expression analysis, immune infiltration analysis, correlation analysis, enrichment analysis and single-cell analysis. GEO: Gene Expression Omnibus

OralExplorer is a web-based tool designed for research on oral diseases (Fig. 1). It consists of five main analysis function modules. These modules allow users to explore the results of differential gene expression analysis, immunoinfiltration analysis, correlation analysis, pathway enrichment analysis and single-cell analysis. Users can also customize the parameters of the analysis methods to suit their specific needs. Additionally, the tool provides detailed analysis results and high-resolution visualization images that can be easily accessed and downloaded for local use. The "Home" page offers a general overview of OralExplorer's structure, accompanied by sample visualizations of each analysis module. The "Docs" page provides contact information for the web developer as well as answers to frequently asked questions about using the website and its solutions.

### Data summary

OralExplorer incorporates 35 datasets of human oral inflammatory diseases across 6 main oral disease types, including peri-implantitis, periodontitis, pulpitis, caries, temporomandibular joint osteoarthritis and gingivitis. These datasets consist of a total of 901 samples. The input data module provides a table summarizing information about each dataset, including disease type, species, sample size, and more. This table also includes an integrated summary and design overview of the dataset, making it easy to access detailed experimental information. Users can simply click on the hyperlinks within the table to navigate to the corresponding GEO dataset webpage if they wish to explore a specific dataset further.

### Differential gene expression analysis

**Fig. 2:**
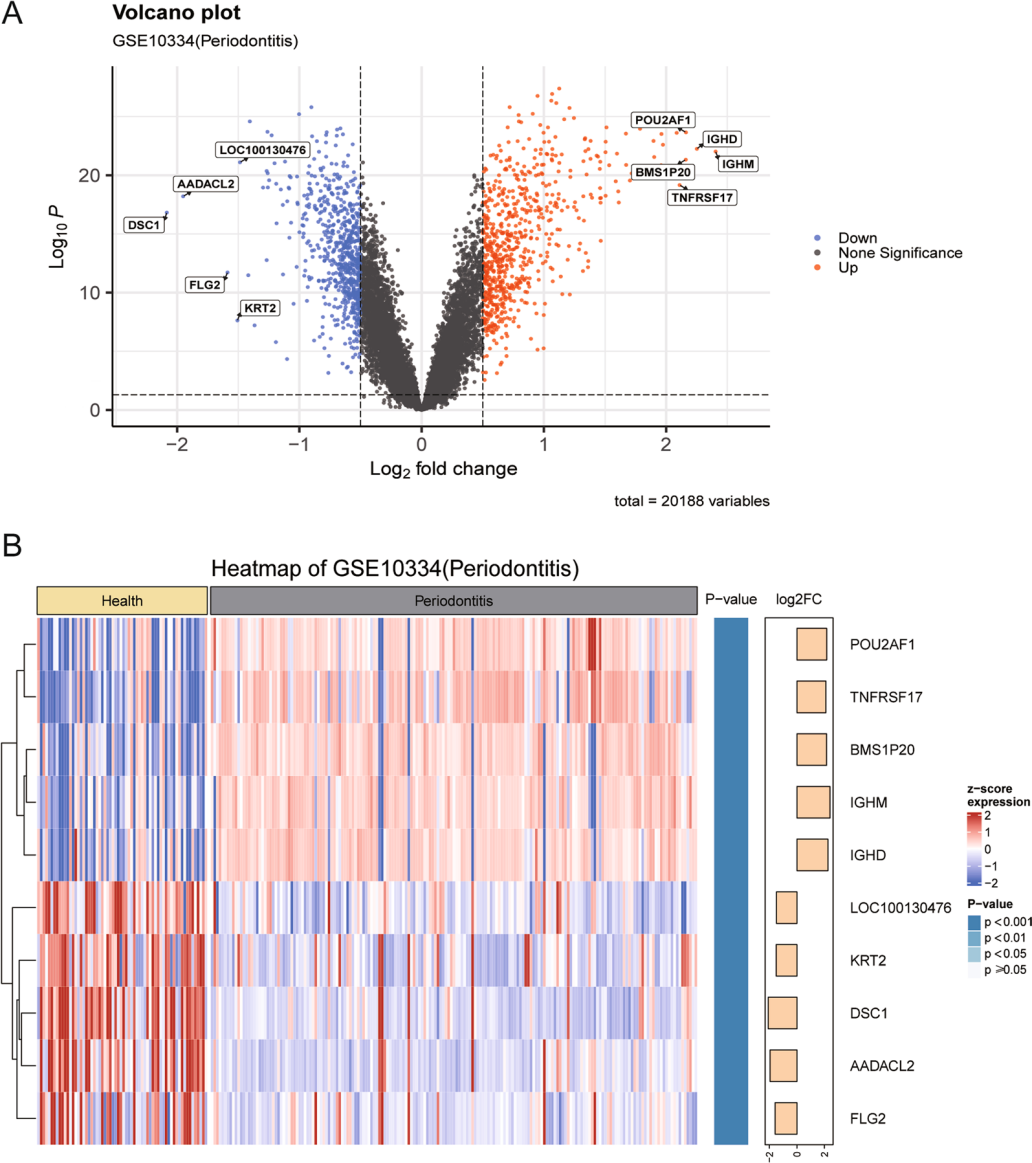
Differentially expressed gene analysis. **A** The results of gene variance analysis were visualized by volcano plots. The red points represent upregulated genes, the blue points represent downregulated genes, and the grey points represent genes that are not statistically significant. **B** Gene difference analysis results visualized by heatmap. The top of the heatmap shows the data groupings (healthy and diseased groups). The body of the heatmap displays the normalized gene expression values with squares of different colour gradients. Darker red colours represent higher gene expression values, and darker blue colours represent lower gene expression values. The right side of the heatmap shows the significance (p value) and fold change (Log2FoldChange) of the results of differential gene expression analysis. The calculation of p values and fold changes was based on the limma package

For the results of differential gene expression analysis between disease group samples and normal group samples, two visualization options are provided to users: volcano plots and heatmaps (Fig. 2). The default significance thresholds for differentially expressed genes are a p value less than 0.05 and an absolute log2Foldchange value greater than 1.5. By default, the results display 10 gene names that are significantly upregulated and downregulated, which are annotated in the visualization results. However, users have the flexibility to adjust the significance thresholds and gene name annotations according to their individual analysis and visualization needs. Additionally, users can review the complete differential expression analysis results online on the table page or download and save the results locally. OralExplorer serves as a valuable tool for researchers to obtain further support for their hypotheses. For instance, Yu [36] conducted protein blotting experiments, immunohistochemical analysis, and real-time qPCR on gingival tissues obtained from patients with periodontitis. The findings revealed that the expression levels of VNN1 and VNN2 were significantly upregulated in the periodontitis cohort. These observations align with the results of the differential expression analysis of GSE10334-Periodontitis in OralExplorer, as shown in Fig. 2A, B.

### Immune infiltration analysis

**Fig. 3:**
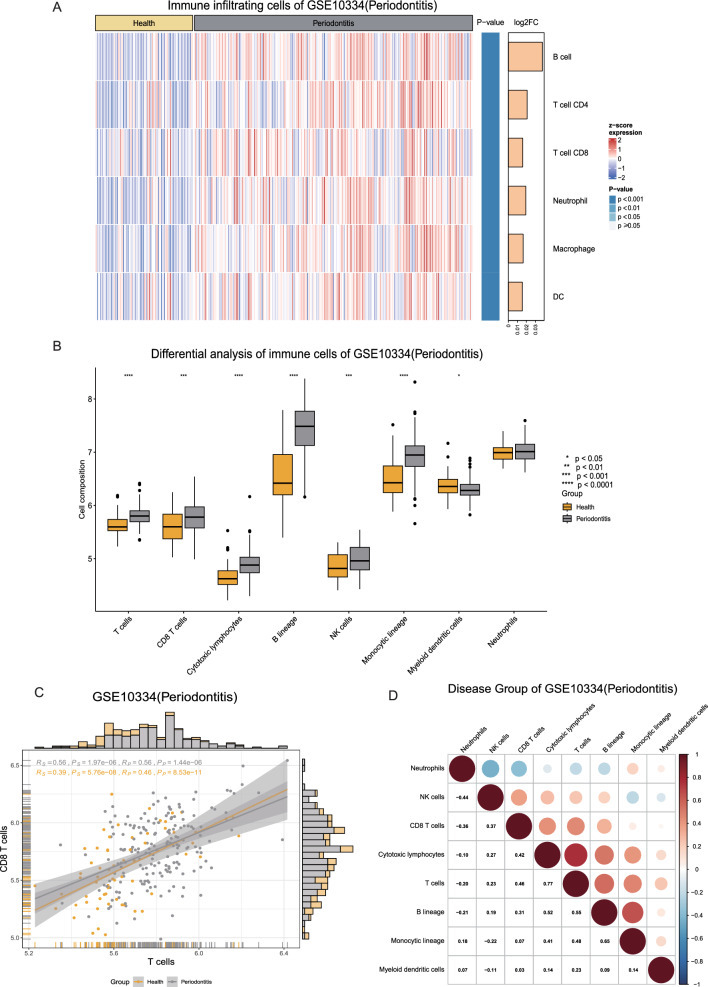
Immune infiltration analysis. **A** Heatmap of differences in immune infiltration between the disease and healthy groups. The top of the heatmap shows the data grouping (healthy and disease groups). The main body of the heatmap shows the normalized immune infiltration scores in squares with different colour gradients. Darker red indicates more infiltration of that immune cell, and darker blue indicates less infiltration of that immune cell. The significance (p value) and fold change (Log2FoldChange) values for the results of the immune infiltration difference analysis are shown on the right side of the heatmap. The calculation of p values and fold changes was based on the limma package. **B** Box plots of immune infiltration differences. Yellow and gray represent healthy and diseased groups, respectively. “*” indicates the statistical results for immune cell differences. *p < 0.05, **p < 0.01, ***p < 0.001, and ****p < 0.0001. **C** Scatterplot of the correlations between two immune cells. Rs and Ps represent Spearman's correlation coefficients and p values, respectively. Rp and Pp represent Pearson's correlation coefficients and p values, respectively. **D** Heatmap of the correlation between multiple immune cells, where the numbers in each grid are the corresponding correlation coefficients. Red represents a positive correlation, and blue represents a negative correlation. The darker the colour, the stronger the correlation

Immune cell composition and proportions play a significant role in disease regression [37]. To characterize the composition and proportions of immune cells in oral diseases, we employed six advanced immune cell algorithms: TIMER, xCell, CIBERSORT, EPIC, quanTIseq, and MCPcounter. We performed an analysis to identify differences in immune infiltration between various groups, and the outcomes were visualized in heatmaps or boxplots, as shown in Fig. 3A and B. Furthermore, in OralExplorer, users can explore the correlations between different immune cell infiltration profiles, including correlations between two or more immune cells, as illustrated in Fig. 3C and D.

### Correlation analysis

**Fig. 4:**
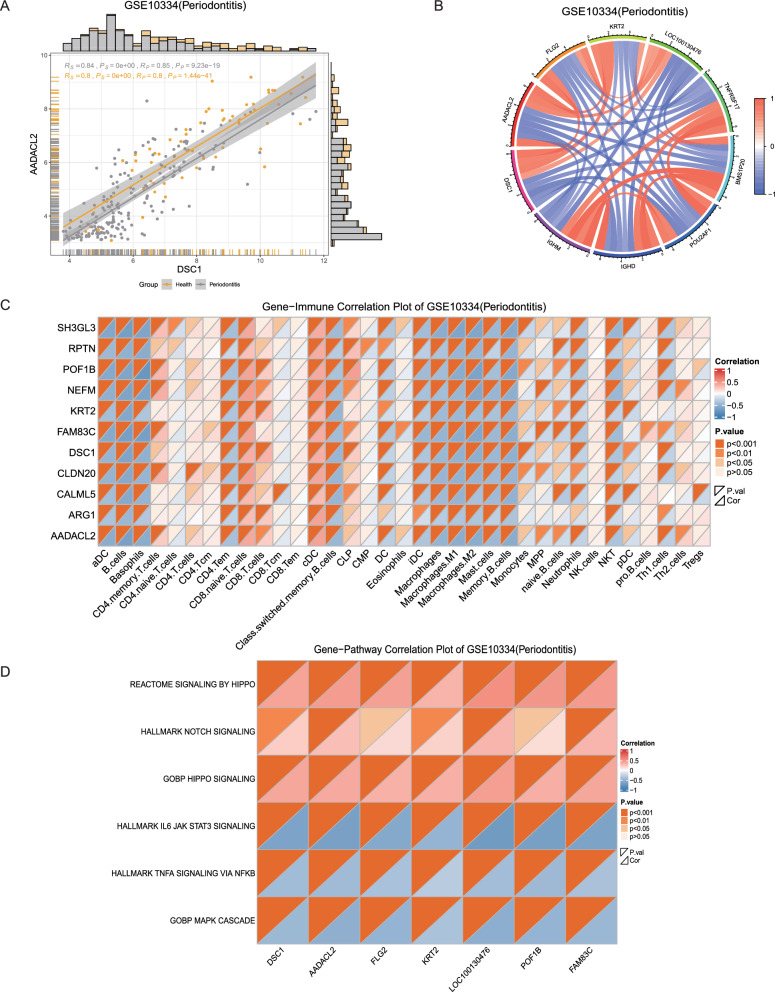
Correlation analysis. **A** Scatterplot of correlations between single genes showing the correlations between 2 genes. Rs and Ps represent Spearman correlation coefficients and p values, respectively, and Rp and Pp represent Pearson correlation coefficients and p values, respectively. **B** Correlation chord plots between multiple genes. The colour of the line connecting the genes represents the magnitude of the correlation between the two genes. Red and blue represent positive and negative correlations, respectively. The darker the colour, the stronger the correlation. **C** Heatmap of gene-immune cell correlation. The red and blue colours in the heatmap represent the normalized correlation results. The darker the red colour is, the stronger the positive correlation, and the darker the blue colour is, the stronger the negative correlation. Missing values are shown in white. The colour shade of the orange triangles in the upper left corner of each box indicates the statistical results for correlation differences, where light to dark orange triangles represent p > 0.05, p < 0.05, p < 0.01, and p < 0.001, respectively. **D** Gene-pathway correlation heatmap showing the correlation between selected genes and selected pathways. Red and blue in the heatmap represent positive and negative values, respectively, of normalized correlations; the darker the red colour is, the stronger the positive correlation, and the darker the blue colour is, the stronger the negative correlation. Missing values are shown in white. The orange triangles in the upper left corner represent different p values according to the colour shade, where light to dark orange represents p > 0.05, p < 0.05, p < 0.01, and p < 0.001, respectively

Genes, pathways, and cells exhibit interconnectedness and interact with each other. To delve deeper into these associations, we offer users the ability to conduct correlation analyses involving genes, immune cells, and pathways. Users can conveniently search for specific disease types and gene sets that they are interested in, filter subgroups for inclusion in the samples, and select target genes, immune cells, or pathways. Our platform provides two correlation analysis algorithms (Spearman and Pearson) as well as various visualization options, such as scatterplots, chord plots, and heatmaps Fig. 4A–D.

### Enrichment analysis

**Fig. 5:**
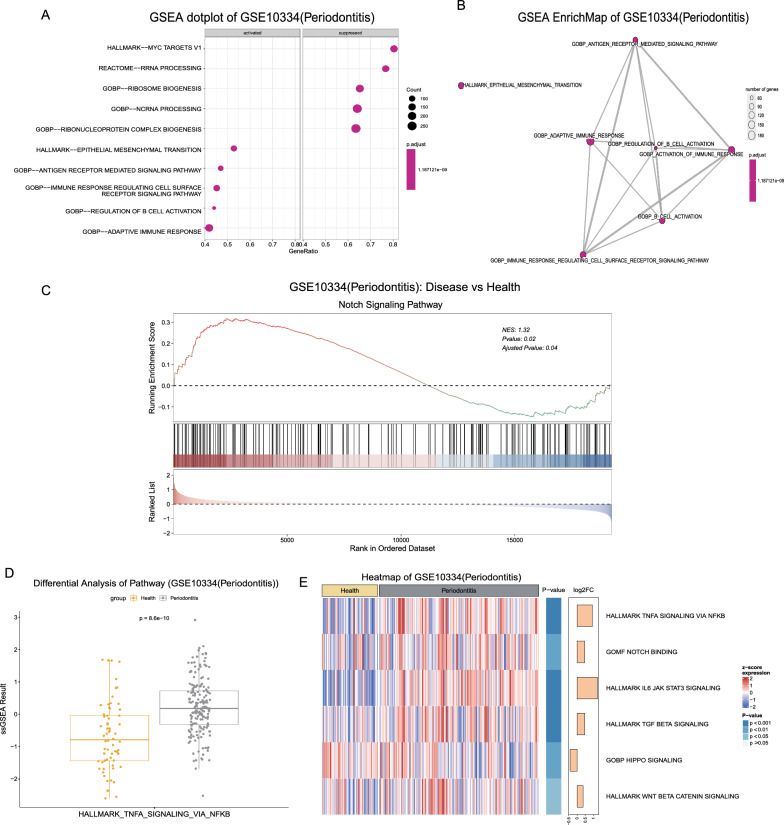
Gene enrichment analysis. **A** Enrichment results of GSEA in the disease group vs. the healthy group are visualized in dotplots. Positive and negative NES values were utilized to differentiate upregulated and suppressed groups. The sizes of the dots represent the numbers of genes enriched in the pathway, and the colour represents the corrected p value. Colours closer to red represent stronger significance, and colours closer to blue represent weaker significance. GeneRatio = Count/setSize. **B** GSEA enrichment map. The size of the circle represents the number of genes enriched in the pathway. The colour represents the size of the adjusted p value, where the closer the colour is to blue, the less statistically significant the difference is, and the closer the colour is to red, the more statistically significant the difference is. **C** GSEA enrichment plot showing the enrichment results of specific pathways in this dataset. **D** ssGSEA was visualized by box plots. Yellow and gray colours were used to represent healthy and diseased groups, respectively, and p values were calculated by the Wilcoxon test. **E** Heatmap visualization of ssGSEA results. The yellow and gray squares at the top represent the healthy and diseased groups, respectively. Red and blue in the body of the heatmap represent positive and negative values of normalized pathway expression, respectively. The darker the red colour, the higher the expression value, and the darker the blue colour, the lower the expression value. The two columns on the right side of the heatmap represent the significance (p value) and the multiplicity of differences (Log2 FoldChange) for each pathway between different groups. The darker the blue colour, the more statistically significant the difference. The longer the yellow columns are, the greater the multiplicity of differences between groups. GSEA: Gene Set Enrichment Analysis; NES: normalized enrichment score; ssGSEA: Single-sample Gene Set Enrichment Analysis

OralExplorer offers two commonly used pathway enrichment algorithms, GSEA and ssGSEA, along with 13,661 commonly used pathway gene sets from the MSigDB database. Users have the option to choose from three visualization methods, dotplot, Enrichmap, and classic GSEA enrichplot, to present the results of GSEA pathway enrichment analysis (Fig. 5A–C). For ssGSEA, users can select specific pathways of interest to generate boxplots illustrating the differences in pathway scores between normal and disease groups (Fig. 5D). Additionally, users can choose up to 15 pathways to create heatmap visualizations of ssGSEA pathway enrichment analysis results (Fig. 5E). Publication-worthy visualizations and detailed enrichment analysis results can be downloaded by users for further study.

### Single-cell analysis

**Fig. 6:**
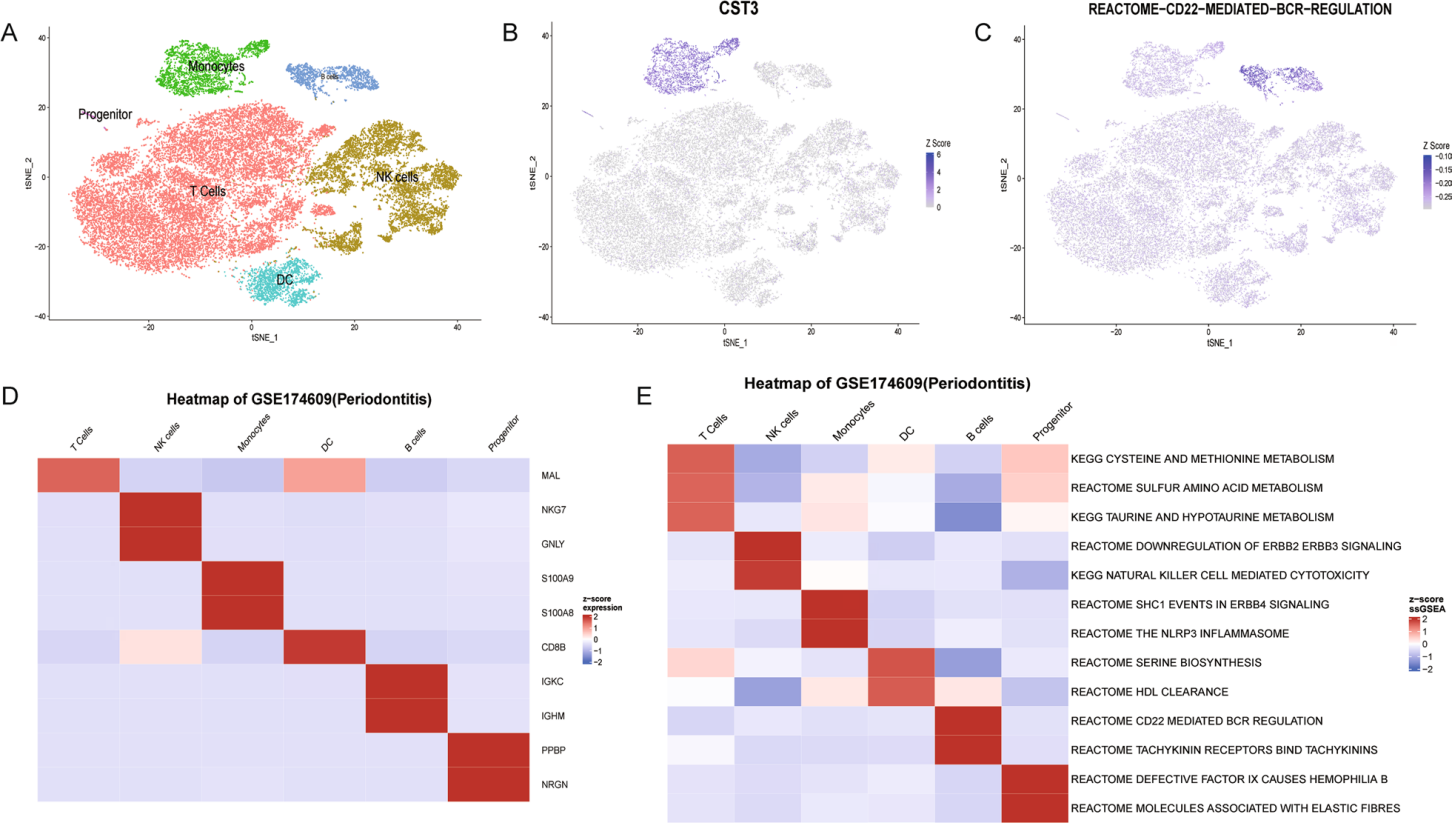
Single-cell analysis. **A** Single-cell clustering map obtained after dimensionality reduction using PCA and TSNE on the oral single-cell dataset and manual annotation of cell clusters. **B** Gene feature maps: feature maps of the expression values of genes normalised to be displayed in different cells. **C** Pathway feature map: ssGSEA score expression values in different cells. **D** Heatmap of expression values of cells after normalisation in different cluter. **E** Heatmap of normalised ssGSEA scores in different cluter. ssGSEA: Single-sample Gene Set Enrichment Analysis

In OralExplorer, single-cell analysis visualizations encompass cluster plot, feature plot, and heatmap visualizations. The cluster analysis visualization displays the oral single-cell dataset post-downscaling and clustering, enabling users to easily observe the result of cell clusters (Fig. 6A). Furthermore, we calculated ssGSEA scores() for individual cells and clusters for subsequent visualization in feature plots and heatmaps, respectively. The feature plot allows users to select the gene or pathway of interest and observe its expression in different cells (Fig. 6B, C). Similarly, the heatmap enables users to readily observe the expression of various genes and pathways across different clusters (Fig. 6D, E).

### Experimental validation: investigation of differentially expressed genes related to periodontitis

**Fig. 7:**
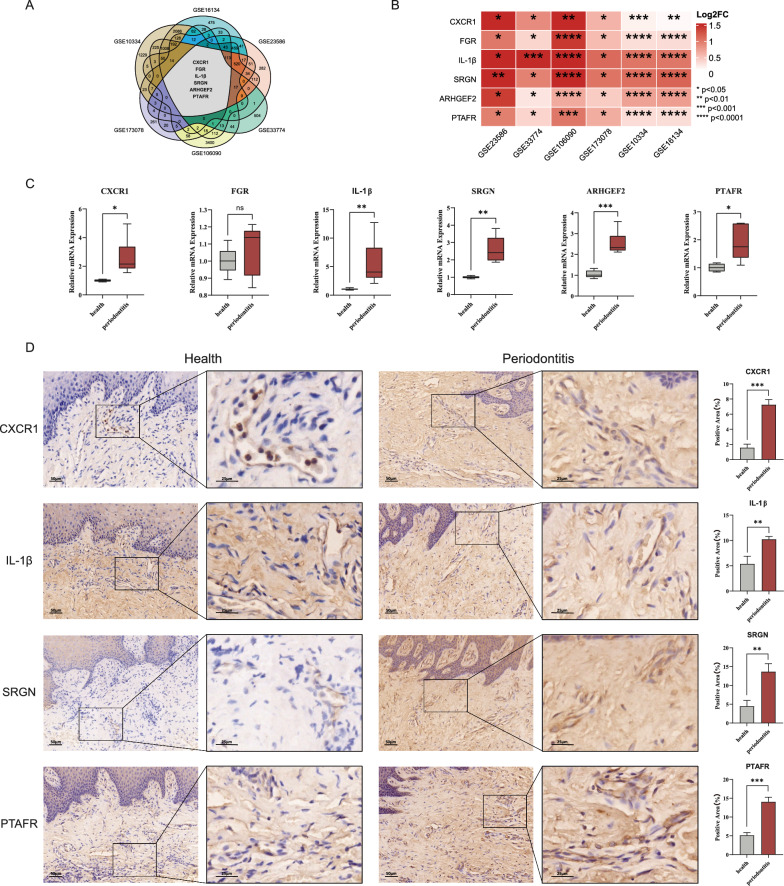
Validation of periodontitis-associated differentially expressed genes using qPCR and IHC. **A** Venn diagram shows the intersection of differentially expressed genes in the six datasets, including IL1β, SRGN, CXCR1, FGR, ARHGEF2, and PTAFR. **B** Heatmap illustrates differentially expressed genes in the six datasets. **C** Boxplots show the qPCR results of the six genes (IL1β, SRGN, CXCR1, FGR, ARHGEF2, and PTAFR) in gingival tissues of the periodontitis group and healthy group. **D** IHC result plots and corresponding histograms show the protein expression levels of IL1β, SRGN, CXCR1 and PTAFR in gingival tissues of periodontitis and healthy groups. *p < 0.05, **p < 0.01, ***p < 0.001, ****p < 0.0001. IHC: Immunohistochemistry; qPCR: quantitative polymerase chain reaction

We conducted online differential gene expression analysis using OralExplorer on multiple datasets associated with periodontitis, including GSE10334, GSE16134, GSE23586, GSE33774, GSE173078, and GSE106090. The analysis revealed simultaneous significant upregulation of IL1β, SRGN, CXCR1, FGR, ARHGEF2, and PTAFR in all six periodontitis-related datasets (Fig. 7A, B, Additional file 4: Table S4). To validate the expression levels of these genes, we conducted qPCR on human periodontitis patient samples and normal human periodontal samples. Except for FGR, all other genes exhibited significant upregulation in the periodontitis group (Fig. 7C). Additionally, we conducted immunohistochemical analysis to explore the protein translation of IL1β, SRGN, CXCR1, and PTAFR in patients with periodontitis. Similar to the mRNA expression results, enhanced protein expression of IL1β, SRGN, CXCR1, and PTAFR was observed in the periodontitis group (Fig. 7D).

## Discussion

Because of the widespread use of sequencing technology, an increasing number of oral disease-related datasets are now publicly available. This availability has greatly facilitated the exploration of oral disease development mechanisms by dentists and dental researchers. However, dentists without a programming background still face significant challenges in utilizing these datasets for in-depth analysis and visualization. To address this issue, we present OralExplorer, a user-friendly, interactive web tool specifically designed for the analysis of oral datasets. OralExplorer integrates 35 human oral inflammatory disease-related datasets from the GEO dataset, consisting of 901 samples across six main oral disease types. It offers five major analysis modules: differential gene expression analysis, immune infiltration analysis, correlation analysis, pathway enrichment analysis and single-cell analysis. OralExplorer provides a simple and intuitive visualization interface as well as user-friendly operations, enabling dentists and medical researchers to effectively explore the value of oral disease data.

OralExplorer is a user-friendly web tool for oral disease research that offers unique advantages in terms of data inclusion, interface and operation, analysis functions, and customization features. First, OralExplorer fills a significant gap in zero-code online analysis in the dental field by combining the bioinformatics analysis of oral-related disease with emerging Shiny web tools. This integration allows researchers to conduct preliminary studies of oral disease mechanisms through simple online operations, even without programming knowledge. Moreover, the interface of OralExplorer is designed to be simple and clear, with user-friendly prompts, further improving the ease of use. In terms of analysis functions, OralExplorer harnesses transcriptome data to provide various analysis capabilities. These include exploring gene expression differences at the transcriptional level, pathway enrichment analysis, correlation analysis, immune infiltration analysis and single-cell analysis. Within the immune infiltration analysis module, OralExplorer offers six reliable immune infiltration algorithms, allowing users to assess the immune infiltration of samples and generate or validate hypotheses related to inflammation and immune cells. The ability to compare results from multiple immune infiltration algorithms in OralExplorer enhances the reliability of the conclusions. Finally, OralExplorer offers a wealth of customization features to cater to different research needs. Users have the flexibility to adjust analysis parameters, analysis methods, and visualization methods according to their requirements. Additionally, we compared OralExplorer with many popular web-based tools, such as HPV-TIMER [38] and CAMOIP [39]. Our findings revealed that OralExplorer not only possesses comparable functionality to these tools but also undergoes validation using qPCR and immunohistochemistry results from clinical samples, ensuring the scientific robustness and reliability of OralExplorer analyses. Overall, OralExplorer’s unique combination of datva inclusion, interface and operation, analysis functions, and customization features make it a valuable tool for oral disease research.

To validate the accuracy and reliability of the online analysis provided by OralExplorer, we employed additional validation methods, such as qPCR and IHC. An online differential expression analysis conducted using OralExplorer revealed several genes that were significantly upregulated in all six periodontitis-related datasets, including IL-1β, SRGN, CXCR1, FGR, ARHGEF2, and PTAFR. These results align with our own laboratory findings. Specifically, we observed that IL-1β, SRGN, CXCR1, and PTAFR exhibited significant upregulation at both the RNA transcript and protein translation levels in periodontitis patients compared to healthy periodontal patients. We also observed a significant increase in ARHGEF2 expression in periodontitis patients. Notably, the findings we obtained are consistent with previous studies conducted by other researchers. For instance, Cheng et al. demonstrated that IL-1β expression is upregulated in periodontitis and plays a role in inflammation, immunomodulation, and bone resorption in periodontitis [40]. Additionally, Cai et al. revealed that FGR expression is upregulated in periodontitis and may serve as an important biomarker of the condition [41]. Caetano et al. conducted single-cell transcriptional profiling on gingival tissues obtained from healthy individuals and patients with periodontitis [42]. The analysis revealed a notable increase in CD2 expression in T-cells, a finding that was subsequently validated using OralExplorer.

However, OralExplorer has certain limitations. Currently, we do not have sufficient resources to include histologic information, and there is a lack of diversity in terms of the included disease types. Furthermore, our database only includes data on human species, without any information on animal models. In the future, we plan to address these shortcomings by continuously updating OralExplorer to incorporate more oral disease-related datasets. Additionally, 2 weeks prior to each OralExplorer update, an announcement is posted on our website to remind users to conduct and save the necessary analyses in a timely manner. Our goal is to enhance the tool and better support oral inflammatory disease-related biomarker research.

## Conclusions

OralExplorer is an efficient user-friendly web tool for analysing oral disease biomarkers. It enables oral researchers to explore transcriptome data related to oral diseases in public databases with maximum efficiency. We welcome feedback from users and will continue to update the website to ensure its usefulness as an auxiliary tool for oral disease research in the future.

### Supplementary Information

**Additional file 1: Table S1.** The details of 35 datasets that were relevant to our study on oral inflammatory diseases.

**Additional file 2: Table S2.** Immune cell types incorporated in different immune infiltration analysis methods.

**Additional file 3: Table S3.** Pathways involved in GSEA and ssGSEA, including 50 hallmark pathways, 3050 C2 canonical pathways, and 10,561 C5 gene ontology pathways

**Additional file 4: Table S4.** Results of differential gene analyses in multiple datasets associated with periodontitis.

## Data Availability

The datasets supporting the conclusions of this article are available in the Gene Expression Omnibus repository, https://www.ncbi.nlm.nih.gov/geo/.

## Abbreviations

DEG: Differentially expressed genes
DEPC: Diethylcarbamoyl pyrocarbonate
GSEA: Gene Set Enrichment Analysis
NES: Normalized enrichment score
GEO: Gene Expression Omnibus
IHC: Immunohistochemistry
PCA: Principal component analysis
qPCR: Quantitative polymerase chain reaction
ssGSEA: Single-sample Gene Set Enrichment Analysis
t-SNE: T-distributed Stochastic Neighbor Embedding
